# 10-year trends in noncommunicable disease mortality in the Caribbean region

**DOI:** 10.26633/RPSP.2019.37

**Published:** 2019-03-27

**Authors:** Hilda Razzaghi, Damali N. Martin, Sarah Quesnel-Crooks, Yuling Hong, Edward Gregg, Glennis Andall-Brereton, Vilma Gawryszweski, Mona Saraiya

**Affiliations:** 1 U.S. Centers for Disease Control and Prevention U.S. Centers for Disease Control and Prevention AtlantaGeorgia United States of America U.S. Centers for Disease Control and Prevention, Atlanta, Georgia, United States of America.; 2 National Cancer Institute National Cancer Institute RockvilleMaryland United States of America National Cancer Institute, Rockville, Maryland, United States of America.; 3 Caribbean Public Health Agency Caribbean Public Health Agency Port-of-Spain Trinidad and Tobago Caribbean Public Health Agency, Port-of-Spain, Trinidad and Tobago.; 4 Pan American Health Organization Pan American Health Organization WashingtonD.C. United States of America Pan American Health Organization, Washington, D.C., United States of America.

**Keywords:** Mortality, noncommunicable diseases, cardiovascular diseases, neoplasms, diabetes mellitus, Caribbean Region, Guyana, Suriname, Mortalidad, enfermedades no transmisibles, enfermedades cardiovasculares, neoplasias, diabetes mellitus, Región del Caribe, Guyana, Suriname, Mortalidade, doenças não transmissíveis, doenças cardiovasculares, neoplasias, diabetes mellitus, Região do Caribe, Guiana, Suriname

## Abstract

**Objective.:**

Between 2006 and 2016, 70% of all deaths worldwide were due to noncommunicable diseases (NCDs). NCDs kill nearly 40 million people a year globally, with almost three-quarters of NCD deaths occurring in low- and middle-income countries. The objective of this study was to assess mortality rates and trends due to deaths from NCDs in the Caribbean region.

**Methods.:**

The study examines age-standardized mortality rates and 10-year trends due to death from cancer, heart disease, cerebrovascular disease, and diabetes in two territories of the United States of America (Puerto Rico and the U.S. Virgin Islands) and in 20 other English- or Dutch-speaking Caribbean countries or territories, for the most recent, available 10 years of data ranging from 1999 to 2014. For the analysis, the SEER*Stat and Joinpoint software packages were used.

**Results.:**

These four NCDs accounted for 39% to 67% of all deaths in these 22 countries and territories, and more than half of the deaths in 17 of them. Heart disease accounted for higher percentages of deaths in most of the Caribbean countries and territories (13%-25%), followed by cancer (8%-25%), diabetes (4%-21%), and cerebrovascular disease (1%-13%). Age-standardized mortality rates due to cancer and heart disease were higher for males than for females, but there were no significant mortality trends in the region for any of the NCDs.

**Conclusions.:**

The reasons for the high mortality of NCDs in these Caribbean countries and territories remain a critical public health issue that warrants further investigation.

## RESUMEN

**Objetivo.:**

Entre los años 2006 y 2016, las enfermedades no transmisibles (ENT) ocasionaron un 70 % de todas las muertes mundiales. Las ENT son responsables de la muerte de aproximadamente 40 millones de personas al año a nivel mundial, de las cuales casi tres cuartas partes tienen lugar en países de ingresos medianos y bajos. El objetivo de este estudio es evaluar las tasas de mortalidad y las tendencias relacionadas con las defunciones por ENT en el Caribe.

**Métodos.:**

En el estudio se examinan las tasas de mortalidad ajustadas por edad y las tendencias a lo largo de diez años relacionadas con la muerte por cáncer, cardiopatías, enfermedades cerebrovasculares y diabetes en dos territorios de Estados Unidos (Puerto Rico e Islas Vírgenes), así como en otros veinte países o territorios de habla inglesa o neerlandesa, empleando la información disponible más reciente que corresponde a los diez años comprendidos entre 1999 y el 2014. Para el análisis, se utilizaron los programas informáticos JointPoint y SEER*Stat.

**Resultados.:**

Estas cuatro ENT representan entre el 39 % y el 67 % del total de muertes en estos 22 países y territorios, y más de la mitad de las muertes en 17 de ellos. Las cardiopatías representan porcentajes mayores de muertes en la mayor parte de los países y territorios del Caribe (13 %-25 %), seguidos por el cáncer (8 %-25 %), la diabetes (4 %-21 %) y las enfermedades cerebrovasculares (1 %-13 %). Las tasas de mortalidad ajustadas por edad relacionadas con el cáncer y las cardiopatías son mayores en hombres que en mujeres, si bien no hubo en la región tendencias significativas relacionadas con la mortalidad en lo que concierne a ninguna ENT.

**Conclusiones.:**

Las causas de la elevada mortalidad por ENT en estos países y territorios del Caribe siguen siendo un grave problema de salud pública que justifica una investigación en profundidad.

## RESUMO

**Objetivo.:**

No período de 2006 a 2016, 70% das mortes na população mundial foram decorrentes de doenças não transmissíveis (DNTs). Cerca de 40 milhões de pessoas morrem por DNTs por ano em todo o mundo, com quase 75% das mortes ocorrendo nos países de baixa e média renda. O objetivo deste estudo foi avaliar as taxas e as tendências de mortalidade por DNTs no Caribe.

**Métodos.:**

Foram examinadas as taxas de mortalidade padronizadas por idade e as tendências ao longo de 10 anos da mortalidade por câncer, doença cardíaca, doença cerebrovascular e diabetes em dois territórios dos Estados Unidos (Porto Rico e Ilhas Virgens Americanas) e em 20 países ou territórios do Caribe de língua inglesa ou holandesa, com base nos últimos dados de 10 anos para o período de 1999 a 2014. Os softwares SEER*Stat e Joinpoint foram usados na análise.

**Resultados.:**

As quatro DNTs estudadas representaram 39% a 67% das causas de mortes nos 22 países e territórios, e foram responsáveis por mais da metade das mortes em 17 deles. A mortalidade na maioria dos países e territórios do Caribe foi maior por doença cardíaca (13% a 25%), seguida do câncer (8% a 25%), diabetes (4% a 21%) e doença cerebrovascular (1% a 13%). As taxas de mortalidade padronizadas pela idade por câncer e doença cardíaca foram maiores nos homens que nas mulheres, mas não se verificaram, na região, tendências de mortalidade significativas para qualquer uma das DNTs.

**Conclusões.:**

A elevada mortalidade por DNTs nos países e territórios do Caribe é ainda um sério problema de saúde pública e os motivos devem ser investigados mais a fundo.

Noncommunicable diseases (NCDs) kill nearly 40 million people a year worldwide, with almost three-quarters of NCD deaths occurring in low- and middle-income countries. Between 2006 and 2016, deaths from NCDs rose by more than 5 million globally, and in 2016 they accounted for more than 70% of all deaths (1). Furthermore, it is predicted that the shared disease burden of NCDs in some of the poorer countries will exceed 80% and will most likely affect younger people (2). Cardiovascular diseases, cancer, and diabetes are among the leading NCDs in the world, accounting for the majority of all NCD deaths (1, 3, 4). The global burden of NCDs is expected to rise further with increases in the global population (especially the older population) and demographic shifts.

The Caribbean region encompasses more than 25 countries and territories that vary in size, geography, resources, and surveillance systems (5). In this region, NCDs carry the highest health-related burdens and are the most common causes of death (6, 7). Aging of the population, successes in primary health care in the treatment of infectious and other diseases, and economic development have led to an NCD-epidemiological transition within the Caribbean region. The proportion of the population of age group 15 years or younger is declining, while young and middle-aged adult age groups (25–44 years and 45–64 years) are increasing (8).

Except for Haiti, the demographic indicators of the Caribbean countries are consistent with the health outcomes that are expected in middle-income countries. This epidemiological transition has implications for increasing the prevalence of noncommunicable diseases such as cardiovascular disease, cerebrovascular disease, diabetes, chronic respiratory diseases, and cancer. The Caribbean region faces the highest burden from NCDs for developing nations in the Americas, and chronic NCDs are the leading cause of death (9). NCDs are linked to more than 70% of deaths in the region, which is similar to the current global average (10).

The increase in the mortality burden from NCDs in the Caribbean region can be due to the rise in risk factors (resulting in an increasing prevalence of NCDs) or the lack of improvement in quality of care and the case-fatality rate. It is vital to reduce the prevalence of NCDs. The World Bank estimates NCDs will have direct and indirect economic impacts on the future health systems and economies of the Caribbean countries, and projects that a reduction in the prevalence of NCDs would lead to positive economic outcomes for the region (10). However, if unaddressed, the prevalence of chronic diseases will continue to have a negative impact on the social and economic well-being of the Caribbean countries (11).

When compared to Canada or the United States of America, the Caribbean countries are more affected by the NCD epidemic. For example, cervical and prostate cancer mortality rates are two to nine times higher in the English- or Dutch-speaking Caribbean than in the United States (12). Mortality from stroke is higher in Haiti, Guyana, and Jamaica than in other countries of Latin America and the Caribbean (13). Guyana, Suriname, and Trinidad and Tobago have the highest prevalence of cardiovascular diseases in the Americas (6, 14, 15). In addition, a study on the burden of diabetes in the Caribbean and North America (7) found a higher age-adjusted prevalence for this disease in most Caribbean-Basin countries than in Canada and the United States. For example, the age-adjusted prevalence of diabetes in Belize and Guyana is approximately twice as high as the age-adjusted prevalence in Canada and the United States.

Although there are sporadic NCD-related mortality reports on various countries in the Caribbean region, a comprehensive regional report of NCD mortality is needed. To characterize NCD mortality patterns, this study examined annual age-standardized mortality rates and 10-year mortality trends due to cancer, heart disease, cerebrovascular disease or stroke, and diabetes for 2 United States territories (Puerto Rico and the U.S. Virgin Islands) and 20 other English- or Dutch-speaking Caribbean countries or territories from 1999 to 2014.

## METHODS

Mortality data for the 20 English- or Dutch-speaking Caribbean countries and territories were obtained from the Caribbean Public Health Agency (CARPHA). Data collection processes in these countries differ from country to country. However, in all of these English- or Dutch-speaking Caribbean countries, medical physicians complete the medical cause of death certificate and trained coders assign codes from the 10th revision of the International Statistical Classification of Diseases and Related Health Problems (ICD-10) to the conditions listed and select the underlying cause of death using World Health Organization (WHO) rules and guidelines. Data submitted to CARPHA (and its predecessor, the Caribbean Epidemiology Centre (CAREC)) are reviewed and validated before data are accepted.

Data for the 2 United States territories (Puerto Rico and the U.S. Virgin Islands) were obtained from the U.S. Centers for Disease Control and Prevention’s National Center for Health Statistics for the same time frame where both mortality and population data were available.

For each of the 22 countries/territories, we calculated age-standardized mortality rates due to cancer (ICD-10: C00-C97), heart disease (ICD-10: I00-I09, I11, I13, I20-I51), cerebrovascular disease (ICD-10: I60-I69), and diabetes (ICD-10: E10-E14). Mortality and population data from the most recent (at the time of analysis) available 10 years (ranging from 1999 to 2014) were used; data were not available for the same 10 years for each of those countries and territories (16).

We also examined whether there were significant 10-year changes in trends of mortality rates for each of the NCDs and each of the countries/territories. Calculations were completed using SEER*Stat software (17) and the WHO 2000-2025 World Standard Population (18). The annual percent change (APC) for trends was calculated using Joinpoint software (19); joinpoint regression analysis was used to assess changes in linear slope for mortality trends over time. Overall trends in mortality were initially assessed with no joinpoints and tested for significant changes in the model with the addition of one point where there is a significant change in the slope of the line (shown as Trend 1 and Trend 2 later in Table 2). The model also computes an estimated APC for each trend by fitting a regression line to the natural logarithm of the rates.

To calculate regional age-standardized mortality rates, we restricted the time period under review to 2005-2010, for which data were available from 18 of the 20 English- or Dutch-speaking Caribbean countries/territories (all of them except the British Virgin Islands and the Turks and Caicos Islands).

Population data were not available from some of the countries/territories for the entire study period. In in these instances, the most recent year of available data (census or mid-year population estimates) was used to populate subsequent years with missing population data. Age-standardized mortality rates are not presented where there were fewer than six deaths annually, an exclusion that has previously been applied with cancer mortality in the Caribbean (12).

## RESULTS

During the period of 1999-2014, four NCDs accounted for 39%-67% of all deaths in the two United States territories and in the other Caribbean countries/territories studied. Those four diseases were also responsible for more than half of the deaths in 17 of the countries/territories. For the combined time period (that is, 10 years for each of the countries/territories), cancer (followed by heart disease) was the leading cause of death for 9 of the countries: Anguilla, Aruba, Barbados, Bermuda, British Virgin Islands, Cayman Islands, Dominica, Jamaica, and Saint Lucia (Table 1).

In the majority of the 20 English- or Dutch-speaking Caribbean countries/territories, heart disease accounted for a higher percentage of deaths (13%-25%) than did cancer (8%-25%), diabetes (4%-21%), and cerebrovascular disease (1%-13%). The U.S Virgin Islands had the highest percentage of deaths due to heart disease (27%) of the 22 Caribbean countries/territories. Among the 20 English- or Dutch-speaking Caribbean countries, the highest percentage of deaths due to heart disease was seen in Montserrat, followed by Bermuda and Trinidad and Tobago. The highest percentages of deaths due to cerebrovascular disease were observed in Saint Kitts and Nevis, Suriname, and Jamaica. The highest percentages of deaths due to diabetes were found in Montserrat, Trinidad and Tobago, and Jamaica. Bermuda, the Cayman Islands, and Aruba had the highest percentages of deaths due to cancer (Table 1).

**TABLE 1:** Cumulative 10-year proportions of death (per 100 000) from four noncommunicable diseases (NCDs) in Puerto Rico, the U.S. Virgin Islands, and 20 other English- or Dutch-speaking Caribbean countries or territories, 1999-2014

Country/Territory		Cancer	Heart disease	Cerebrovascular disease	Diabetes	Cumulative proportions of death due to these four NCDs
English- or Dutch-speaking Caribbean						
	Anguilla (2005-2014)	23.36	17.29	7.17	9.97	57.79
	Antigua and Barbuda (2005-2014)	18.43	20.90	7.39	8.91	55.63
	Aruba (2005-2014)	23.79	20.11	8.22	6.05	58.16
	Bahamas (2004-2013)	17.91	20.70	7.11	5.16	50.88
	Barbados (2004-2013)	21.16	16.53	8.44	9.07	55.20
	Belize (2005-2014)	11.58	13.04	5.63	8.87	39.11
	Bermuda (2005-2014)	26.64	23.46	7.70	5.17	62.97
	British Virgin Islands (1999-2004; 2006; 2008-2010)	19.88	13.93	1.43	5.23	40.47
	Cayman Islands (2004-2013)	25.19	17.88	3.97	3.90	50.94
	Dominica (2005-2014)	19.67	19.53	10.61	7.87	57.68
	Grenada (2005-2014)	19.33	20.28	10.40	10.08	60.09
	Guyana (2004-2013)	8.29	21.02	10.93	8.11	48.35
	Jamaica (2002-2011)	18.05	14.02	12.27	11.05	55.39
	Montserrat (2005-2014)	13.41	25.05	7.03	21.10	66.59
	Saint Kitts and Nevis (2004-2013)	16.80	17.10	13.47	9.01	56.37
	Saint Lucia (2005-2014)	17.18	16.41	10.42	9.03	53.03
	Saint Vincent and the Grenadines (2005-2014)	15.29	21.15	10.59	9.49	56.51
	Suriname (2005-2014)	12.46	14.48	12.28	6.32	45.54
	Trinidad and Tobago (2001-2010)	13.77	22.29	9.26	13.89	59.21
	Turks and Caicos Islands (2000-2009)	11.89	21.99	2.49	5.81	42.19
U.S. territories						
	Puerto Rico (2005-2014)	17.50	18.37	5.05	10.05	50.97
	U.S. Virgin Islands (2003-2012)	18.39	26.77	5.98	6.31	57.46

***Source:*** Prepared by authors, based on the study results.

**FIGURE 1:**
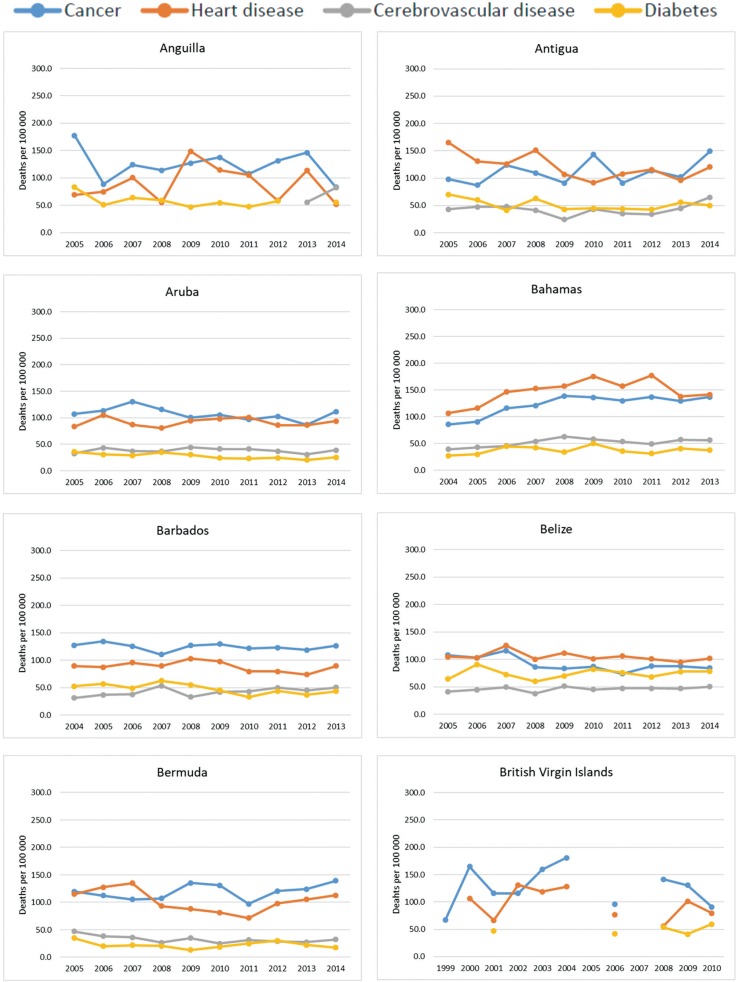

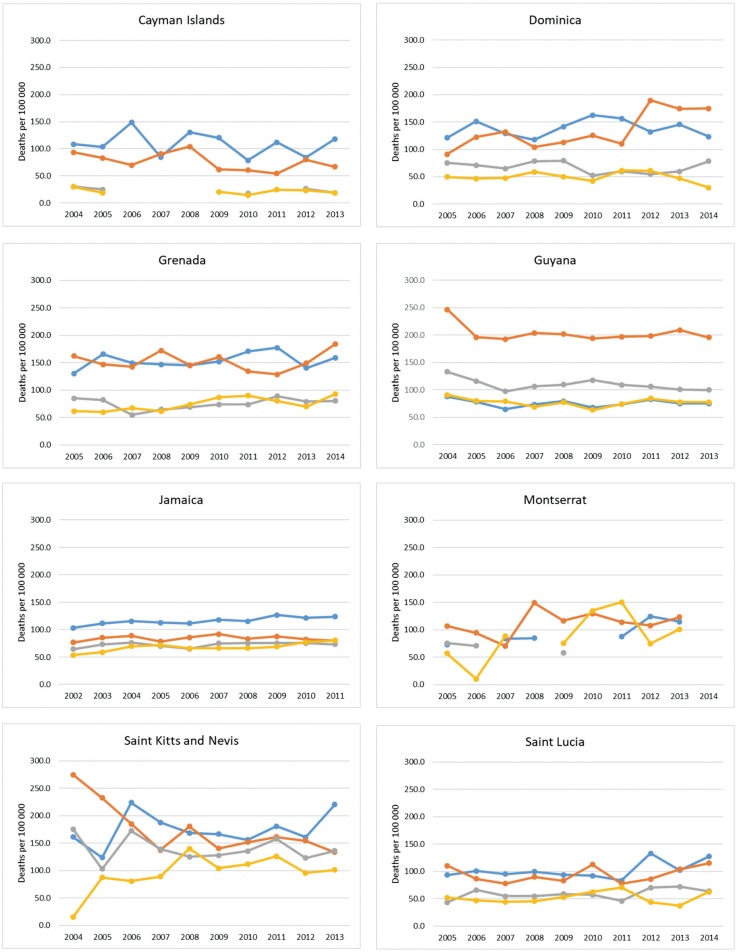

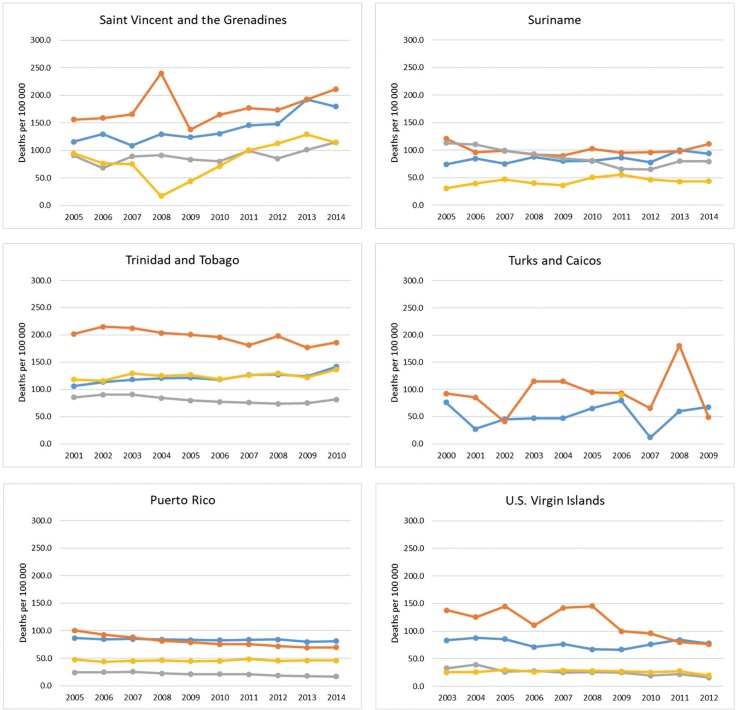
Annual age-standardized mortality rates for cancer, heart disease, cerebrovascular disease, and diabetes in Puerto Rico, the U.S. Virgin Islands, and 20 other English- or Dutch-speaking Caribbean countries and territories, 1999-2014^a^

***Source:*** Prepared by authors, based on the study results.

^a^ Annual rates are only shown where six or more deaths occurred in the given year.

Figure 1 presents annual age-standardized mortality rates (ASMRs) for each of the countries/territories and each of the four NCDs for each of the years with available data. ASMRs for cancer ranged from 64.7 per 100 000 for Guyana in 2006 to 224.0 per 100 000 for Saint Kitts and Nevis in 2006. ASMRs for heart disease ranged from 48.8 per 100 000 for Turks and Caicos in 2009 to 274.8 per 100 000 for Saint Kitts and Nevis in 2004. ASMRs for cerebrovascular disease ranged from 15.2 per 100 000 for Anguilla in 2009 to 175.5 per 100 000 for Saint Kitts and Nevis in 2004. Finally, ASMRs for diabetes ranged from 10.4 per 100 000 for Montserrat in 2006 to 150.2 per 100 000 for Montserrat in 2011.

**FIGURE 2:**
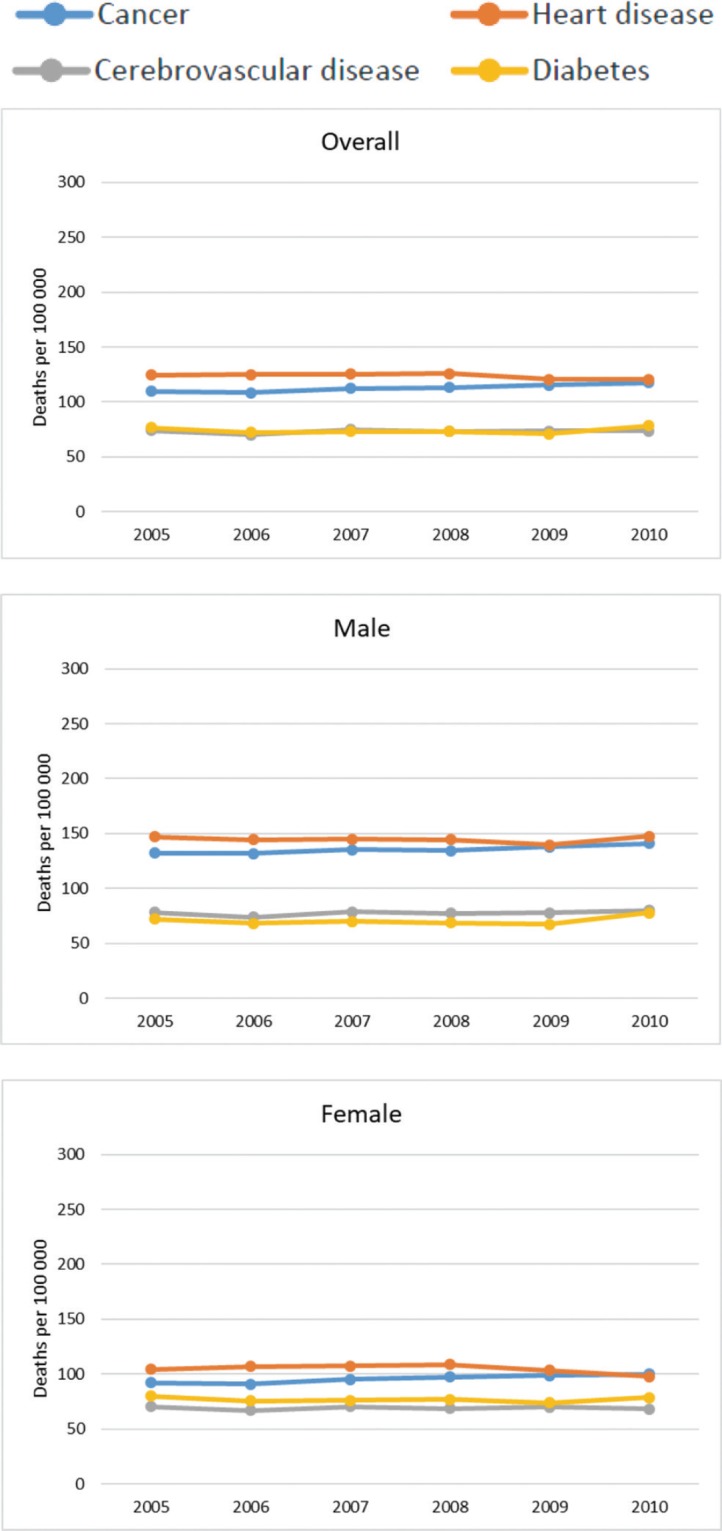
Regional age-standardized mortality rates in 18 English- or Dutch-speaking Caribbean countries/territories, overall and by sex, 2005-2010^a^

***Source:*** Prepared by authors, based on the study results.

^a^ British Virgin Islands and Turks and Caicos were excluded due to their having fewer than six deaths per year for many years; U.S. Virgin Islands and Puerto Rico were excluded since they are United States territories.

Of the group of 20 English- or Dutch-speaking Caribbean countries/territories, all but two of them (British Virgin Islands and Turk and Caicos Islands) provided mortality data for the years 2005 to 2010, for which regional mortality rates were calculated (Figure 2). Overall, ASMRs for all four NCDs remained consistent in this five-year period. However, the gap in mortality between cancer and heart disease closed as the years progressed. When examining regional mortality rates by sex, ASMRs due to cancer and heart disease ranged, respectively, from 131.6 to 141.1 and from 139.5 to 147.4 per 100 000 for males, and from 90.7 to 100.0 and from 97.7 to 108.6 per 100 000 for females. ASMRs due to cerebrovascular disease and diabetes ranged, respectively, from 73.9 to 79.8 and from 68.0 to 77.7 per 100 000 for males, and from 66.7 to 70.5 and from 74.0 to 80.0 per 100 000 for females. Thus, there were no substantial changes in mortality trends in the region from 2005 to 2010 for any of the NCDs (Figure 2).

Investigation into 10-year NCD mortality trends by country/territory revealed some significant trends, as noted in Table 2. (For the majority of the countries/territories, trends remained unchanged over the 10-year study period, thus no values are presented for Trend 2 in those cases in Table 2.). For example, cancer mortality significantly increased in the Bahamas (2004-2008), Jamaica (2002-2011), Saint Vincent and the Grenadines (2005-2014), Suriname (2005-2014), and Trinidad and Tobago (2001-2010). Significant decreases were observed in Belize (2005-2014) and Puerto Rico (2005-2014). Mortality due to heart disease significantly increased in the Bahamas (2004-2009) and Dominica (2005-2014), but significantly decreased in Antigua and Barbuda (2005-2014), Bermuda (2005-2011), Saint Kitts and Nevis (2004-2007), Trinidad and Tobago (2001-2010), Puerto Rico (2005-2014), and the U.S. Virgin Islands (2003-2012). Mortality due to cerebrovascular disease increased significantly in Anguilla (2005-2014), the Bahamas (2004-2008), and Saint Vincent and the Grenadines (2005-2014), but significantly decreased in Bermuda (2005-2014), Suriname (2005-2012), Trinidad and Tobago (2001-2010), Puerto Rico (2005-2014), and the U.S. Virgin Islands (2003-2012). Mortality due to diabetes significantly increased in Grenada (2005-2014) and Jamaica (2002-2011) but decreased in Aruba (2005-2014) and Barbados (2004-2013).

## DISCUSSION

Cancer, cardiovascular disease, and diabetes accounted for more than half the mortality in the English- or Dutch-speaking Caribbean countries/territories in the study. The reasons for the high mortality due to NCDs in those countries/territories remains a critical public health issue that warrants further investigation.

NCD-associated risk factors such as tobacco smoking, harmful use of alcohol, poor diet, and physical inactivity substantially affect NCD mortality (20). Physical inactivity has been associated with an increased risk of certain NCDs, including heart disease, diabetes, and cancer. Available data shows that 31% of the world’s population are not meeting the minimum recommendations for physical activity (21). In 2010, the global prevalence of inactivity in adults aged 18 years and older was 23%. Of the WHO regions, the Region of the Americas (which includes the Caribbean region) had one of the highest prevalence levels of insufficient physical inactivity, at 32% (22).

Harmful use of alcohol has been estimated to be responsible for 3.3 million deaths globally every year, which represent 5.9% of all deaths (23). The highest alcohol consumption levels are found in the developed world, in particular in the WHO European Region and the Region of the Americas. Total alcohol consumption per capita among those 15 years of age and older in the Caribbean countries was more than 7.5 liters of pure alcohol in 2010 (23). Globally, obesity rates have more than doubled since 1980. WHO estimated that in 2014 more than 600 million adults worldwide (~13%) over the age of 18 were obese (22).

Among world regions, the Region of the Americas has one of the highest proportions of adults who are obese, at more than 30%. Based on 2014 data, the Bahamas (36.2%) surpassed the United States (33.7%) (22). Jamaica’s obesity rates are above 30% in those 15 years and older (24).

Latin American and Caribbean nations have a high burden of cardiovascular disease, with 8.7% of total cardiovascular disease deaths being due to high sodium intake (25). A 30% relative reduction in mean population intake of salt/sodium is one of nine global targets for NCDs for 2025 that have been set by WHO (26). PAHO and civil society in Latin America and the Caribbean have taken action to reduce sodium intake, such as with social marketing strategies to reduce salt consumption (27-29).

The findings of our study underscore the importance of implementing evidence-based policies and programs that could mitigate chronic disease risk factors in the Caribbean region (30). Primary health care plays an important role for the prevention of obesity among children and adolescents, such as by promoting breast-feeding, healthy dietary habits, and physical activity. A long period of breast-feeding may reduce the prevalence of obesity among children and mothers (31).

There were shifting mortality trends and great variability observed for specific NCDs within the Caribbean region over the time period that we reviewed. Limited data on NCDs in the region have prevented an accurate assessment of the NCD burden and have created challenges in the development of effective interventions. Many of the Caribbean countries and territories lack standardized surveillance systems for NCDs, with a few exceptions. Barbados performs surveillance for stroke, heart disease, and cancer through the Barbados National Registry (BNR) (32). The cost of this population-based NCD surveillance system is around just US$ 1 per person per year, thus making the case that other small island nations with limited resources should consider similar programs for combined surveillance of NCDs (33). In addition, a few Caribbean countries have implemented high-quality population-based registries for the surveillance of cancers (34). The International Agency for Research on Cancer Caribbean Cancer Registry Hub, implemented under the Global Initiative for Cancer Registration, is working to improve the quality of cancer data obtained from Caribbean cancer registries. However, the cost associated with tracking incident cases from these NCDs is relatively high and presents a challenge for the region (35). Nevertheless, a better understanding of the burden of NCDs will aid in the appropriate allocation of resources needed to address these public health concerns. The capacity to address NCDs is a serious issue and should be assessed carefully. One thing that might be useful for that is a new tool that the U.S. Centers for Disease Control and Prevention has developed to help countries identify key opportunities and gaps in NCD capacity; the tool has been piloted in several countries (36).

**TABLE 2:** Joinpoint-software analysis of trends for annual percent change (APC) for cancer, heart disease, cerebrovascular disease, and diabetes mortality in Puerto Rico, the U.S. Virgin Islands, and 19 other English- or Dutch-speaking Caribbean countries/territories, 1999–2014^a^

Country/Territory		Cancer				Hearth disease				Cerebrovascular disease				Diabetes			
		Trend 1		Trend 2^b^		Trend 1		Trend 2		Trend 1		Trend 2		Trend 1		Trend 2	
		Years	APC	Years	APC	Years	APC	Years	APC	Years	APC	Years	APC	Years	APC	Years	APC
English- or Dutch-speaking Caribbean																	
	Anguilla	05-14	-2.00	NA^c^	NA	05-14	0.48	NA	NA	05-14	12.13^d^	NA	NA	05-14	-4.75	NA	NA
	Antigua and Barbuda	05-14	2.95	NA	NA	05-14	-4.02^d^	NA	NA	05-12	-4.77	12-14	38.26	05-14	-2.99	NA	NA
	Aruba	05-14	-1.88	NA	NA	05-14	0.03	NA	NA	05-14	-0.49	NA	NA	05-14	-4.79^d^	NA	NA
	Bahamas	04-08	12.78^d^	08-13	-0.65	04-09	10.13^d^	09-13	-5.76	04-08	10.95^d^	08-13	-1.76	04-13	1.97	NA	NA
	Barbados	04-08	-0.44	NA	NA	04-13	-1.47	NA	NA	04-13	3.76	NA	NA	04-13	-4.27^d^	NA	NA
	Belize	05-14	-3.03^d^	NA	NA	05-14	-1.21	NA	NA	05-14	1.41	NA	NA	05-14	0.54	NA	NA
	Bermuda	05-14	1.57	NA	NA	05-11	-8.52^d^	11-14	14.78	05-14	-4.35^d^	NA	NA	05-14	-2.05	NA	NA
	Cayman Islands	04-13	-1.52	NA	NA	04-13	-4.03	NA	NA	04-06	-45.50	06-13	11.53	04-13	-0.61	NA	NA
	Dominica	05-14	0.57	NA	NA	05-14	6.71^d^	NA	NA	05-14	-1.48	NA	NA	05-12	3.44	12-14	-26.47
	Grenada	05-14	1.24	NA	NA	05-14	0.29	NA	NA	05-07	-16.42	07-14	4.71^d^	05-14	4.34^d^	NA	NA
	Guyana	04-13	-0.34	NA	NA	04-13	-0.96	NA	NA	04-13	-1.85	NA	NA	04-13	-0.62	NA	NA
	Jamaica	02-11	1.67^d^	NA	NA	02-11	0.08	NA	NA	02-11	0.90	NA	NA	02-11	3.12^d^	NA	NA
	Montserrat	05-14	3.29	NA	NA	05-14	-0.79	NA	NA	05-14	-11.25	NA	NA	05-14	4.71	NA	NA
	Saint Kitts and Nevis	04-13	1.49	NA	NA	04-07	-17.26^d^	07-13	-1.05	04-13	-1.53	NA	NA	04-13	4.97	NA	NA
	Saint Lucia	05-14	2.92	NA	NA	05-14	1.27	NA	NA	05-14	2.84	NA	NA	05-14	1.65	NA	NA
	Saint Vincent and the Grenadines	05-14	5.62^d^	NA	NA	05-14	2.01	NA	NA	05-14	2.95^d^	NA	NA	05-14	5.97	NA	NA
	Suriname	05-14	2.18^d^	NA	NA	05-08	-7.84	08-14	2.73	05-12	-7.67^d^	12-14	11.56	05-14	2.62	NA	NA
	Trinidad and Tobago	01-10	2.28^d^	NA	NA	01-10	-1.68^d^	NA	NA	01-10	-1.87^d^	NA	NA	01-10	1.00	NA	NA
	Turks and Caicos Islands	00-09	1.90	NA	NA	00-09	2.58	NA	NA	00-09	NA	NA	NA	00-09	2.91	NA	NA
U.S. territories																	
	Puerto Rico	05-14	-0.63^d^	NA	NA	05-08	-6.79^d^	08-14	-2.78^d^	05-14	-4.31^d^	NA	NA	05-14	0.04	NA	NA
	U.S. Virgin Islands	03-08	-5.01	08-12	4.52	03-12	-6.06^d^	NA	NA	03-12	-7.30^d^	NA	NA	03-12	-1.51	NA	NA

***Source:*** Prepared by authors, based on the study results.

a The British Virgin Islands was excluded from the trends analysis due to missing consecutive years of data.

b A Trend 2 value is available when there was a change in the trend over the respective combined 10 years.

c NA = not available.

d Significantly different from 0 (*P* < 0.05).

In addition to surveillance of NCDs and related risk factors, health sector activities such as screening, diagnosis, and management are also important. Health care systems in many developing countries are not well prepared to deal with a high burden of NCDs, and there is an increased recognition of the need to strengthen primary care services in the Caribbean (37). One strategy that can be used within a primary care setting in the prevention of NCDs is to assess patients for common risk factors and to provide counseling on behavior modification (37). However, a study of primary care performance in Latin America and Caribbean countries identified substantial gaps in primary care, including the lack of health advice offered by physicians to their patients (38). For example, studies done in Caribbean countries confirm that hypertensive and diabetic patients seen in primary care services do not achieve optimal blood pressure and glucose control. These studies indicate that adherence to clinical guidelines for management of hypertension and diabetes may be low among primary care physicians in the Caribbean (39-44). Hypertension is a major risk factor for heart disease and cerebrovascular disease and is also strongly related to diabetes. The Standardized Hypertension Treatment and Prevention Project, which was supported by the U.S. Centers for Disease Control and Prevention and piloted in Barbados, is one example of how it is possible to address the burden of hypertension, by using standardized treatment protocols (45).

In the Caribbean region, NCDs have long been recognized as an area of major concern and one that requires a multisector, regional response (20). One noteworthy move toward that occurred in 2007, with the Caribbean Community’s convening of the world’s first-ever summit of heads of government on NCD prevention and control and the issuance of the landmark Declaration of Port-of-Spain: Uniting to Stop the Epidemic of Chronic NCDs. The 15-point proclamation targets reducing the main NCD risk factors, as well as improving the health service response to NCDs in the region (20). Our analysis in this article is important as a means of monitoring the Caribbean region’s progress towards the Port-of-Spain Declaration targets. Our assessment could also allow individual countries and territories to gauge the success of various strategies that have been implemented to target specific NCDs or their risk factors.

Our study had various limitations that could result in either an under- or overestimation of the presented mortality rates. One limitation was that the denominator data were not available for the entirety of the study years from any of the countries. A second was that trends could not be analyzed for a few of the countries due to either a small number of deaths annually or missing years of data. For example, one country with a small population reported more than 10% “unknown/missing/invalid” cause of death codes, which could compromise the ability to accurately rank the cancer site. A third limitation was that deaths due to diabetes are a problematic index of the level of diabetes care because diabetes is frequently underreported as a cause of death on death certificates. Finally, we were unable to account for the variability that exists among countries regarding the inclusion/exclusion of nonresident deaths in mortality statistics. In addition, the results should be interpreted with caution, given that age-standardized mortality rates may be unreliable in some countries due to small numbers.

Improvements in the collection of health and vital statistics data, along with improvements in systems for surveillance of NCDs, are needed to increase reporting accuracy of regional data for future investigations in the Caribbean.

Despite the various limitations, our results comprehensively highlight country-specific mortality rates and trends for deaths due to NCDs. These findings could be used to inform policy and interventions for strengthening NCD prevention and control in the 2 United States territories and the 20 other English- or Dutch-speaking Caribbean countries and territories that we studied.

The WHO NCD global monitoring framework has nine global NCD targets for 2025, including the reduction of premature mortality from NCDs by 25% (26). Future work to determine whether Caribbean countries and territories are on track to meet this target could be extremely beneficial to the region.

## Author contributions.

All the authors conceived the original idea, guided that analysis, interpreted the results, drafted parts, and reviewed the paper. All the authors reviewed and approved the final version.

## Disclaimer.

The authors hold sole responsibility for the views expressed in the manuscript, which may not necessarily reflect the opinion or policy of the *RPSP/PAJPH* or PAHO. In addition, the findings and conclusions in this report are those of the authors and do not necessarily represent the official position of the U.S. Centers for Disease Control and Prevention.
